# Exosomes secreted by nematode parasites transfer small RNAs to mammalian cells and modulate innate immunity

**DOI:** 10.1038/ncomms6488

**Published:** 2014-11-25

**Authors:** Amy H. Buck, Gillian Coakley, Fabio Simbari, Henry J. McSorley, Juan F. Quintana, Thierry Le Bihan, Sujai Kumar, Cei Abreu-Goodger, Marissa Lear, Yvonne Harcus, Alessandro Ceroni, Simon A. Babayan, Mark Blaxter, Alasdair Ivens, Rick M. Maizels

**Affiliations:** 1 Institute of Immunology and Infection Research, School of Biological Sciences, University of Edinburgh, Edinburgh EH9 3FL, UK; 2 Centre for Immunity, Infection and Evolution, School of Biological Sciences, University of Edinburgh, Edinburgh EH9 3FL, UK; 3 SynthSys, Centre for Synthetic and Systems Biology, School of Biological Sciences, University of Edinburgh, Edinburgh EH9 3BF, UK; 4 Institute of Evolutionary Biology, School of Biological Sciences, University of Edinburgh, Edinburgh EH9 3FL, UK; 5 Laboratorio Nacional de Genómica para la Biodiversidad, Langebio-CINVESTAV, Irapuato 36821, Guanajuato, México; 6 Edinburgh Genomics, School of Biological Sciences, University of Edinburgh, Edinburgh EH9 3FL, UK

## Abstract

In mammalian systems RNA can move between cells via vesicles. Here we demonstrate that the gastrointestinal nematode *Heligmosomoides polygyrus*, which infects mice, secretes vesicles containing microRNAs (miRNAs) and Y RNAs as well as a nematode Argonaute protein. These vesicles are of intestinal origin and are enriched for homologues of mammalian exosome proteins. Administration of the nematode exosomes to mice suppresses Type 2 innate responses and eosinophilia induced by the allergen *Alternaria.* Microarray analysis of mouse cells incubated with nematode exosomes *in vitro* identifies Il33r and Dusp1 as suppressed genes, and Dusp1 can be repressed by nematode miRNAs based on a reporter assay. We further identify miRNAs from the filarial nematode *Litomosoides sigmodontis* in the serum of infected mice, suggesting that miRNA secretion into host tissues is conserved among parasitic nematodes. These results reveal exosomes as another mechanism by which helminths manipulate their hosts and provide a mechanistic framework for RNA transfer between animal species.

## Abstract (web summary)

Mammalian cell-derived exosomes can carry RNA and proteins from cell to cell, but this mode of transport has not been shown in nematodes. Here the authors show that a gastrointestinal parasite secretes exosomes that transfer microRNAs to mammalian cells and regulate innate immunity.

## Graphical abstract

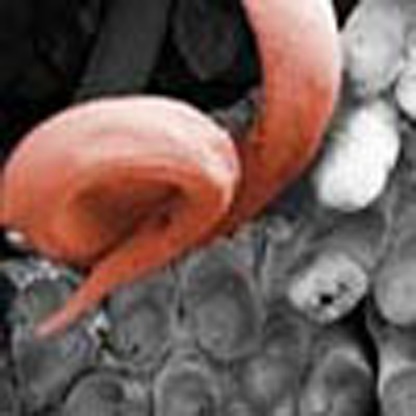

Parasitic nematodes are ubiquitous pathogens of plants and animals, including species that infect over 2 billion people and generally reside in extracellular niches in their hosts. *H. polygyrus* is a parasite related to human hookworm that naturally infects mice, and is in the same nematode clade as *Caenorhabditis elegans*^1^. Within the mouse host, the parasite life cycle is exclusively intestinal: following ingestion, the larvae invade the small intestine, moult into adult worms and emerge into the lumen of the duodenum to mate and to produce eggs expelled in the faeces. The infection induces Type 2 innate and adaptive (Th2) immune responses in parallel with a large expansion of regulatory cells that mediate immunosuppressive effects^2^,^3^,^4^, some of which have beneficial properties in allergy and auto-immunity^5^. Immune suppression has been shown to be mediated in part by a suite of immunomodulatory proteins actively secreted by the nematodes^6^,^7^. Given the burgeoning body of data detailing extracellular small RNAs in mammalian systems, and emerging evidence that these can mediate cell-to-cell communication^8^, it is intriguing to think this mechanism could also be used by parasites. Small RNAs derived from bacteria, plants and parasites have been detected in human body fluids^9^,^10^,^11^,^12^; however, the mechanism by which these are secreted or excreted is unknown, and the meaning of their extracellular existence unclear^13^. We show here that *H. polygyrus* secretes a specific set of miRNAs and full-length Y RNAs that are stabilized against degradation by encapsulation within vesicles. The vesicles are of intestinal origin and are enriched for homologues of mammalian proteins found in exosomes, including heat shock proteins, tetraspanins and ALIX, a protein associated with exosome biogenesis^14^,^15^ as well as a nematode Argonaute (Ago) protein. Local administration of the nematode exosomes to mice by the intranasal route suppresses Type 2 innate responses and eosinophilia induced by the allergen *Alternaria in vivo*. The nematode vesicles are internalized by mouse intestinal epithelial cells *in vitro* and suppress genes involved in inflammation and immunity, including the receptor for the alarmin IL-33 and a key regulator of mitogen-activated protein kinase (MAPK) signalling, DUSP1. This work identifies exosomes as a new class of immunomodulatory complex produced by helminths and provides the first steps towards a mechanistic framework for RNA-mediated communication between animal species.

## Results

### Small RNAs in *H. polygyrus* secretory products

**Figure 1:**
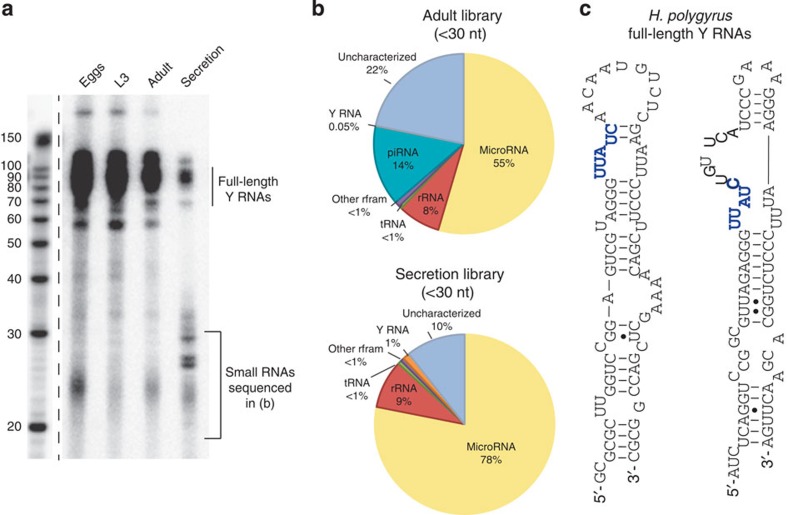
*H. polygyrus* secretory products contain miRNAs and Y RNAs. (**a**) Size distribution of 3′-end labelled (pCp) total RNA extracted from the life stages (1 μg total RNA) or secretion product of *H. polygyrus* (total RNA from equivalent of 15 μg protein secretion product). (**b**) Proportion of *H. polygyrus* small RNA biotypes (<30 nt) identified in sequencing libraries from adult worms and the secretion product. (**c**) Predicted secondary structures of the two families of Y RNA identified in *H. polygyrus* secretion products based on RNAfold, the conserved UUAUC motif is noted in blue.

Total RNA was extracted from the secretory products of *H. polygyrus* and compared with the profile of small RNAs in adult nematodes, eggs and infective larvae. A heterogeneous population of small RNAs <25 nucleotides (nt) was observed in all samples and several additional species between 25 and 30 nt were apparent in the secretion product (Fig. 1a). Small RNA sequencing (<30 nt) identified miRNAs as the dominant class of secreted parasite small RNA (Fig. 1b) and also identified RNA fragments mapping to nematode stem-bulge RNAs, herein referred to as Y RNAs based on their recognized homology to this class of small RNA^16^ (Fig. 1b). In contrast, piRNAs (or ‘21-U’ RNAs) were exclusively identified in the adult library (~14% of reads; Supplementary Fig. 1 and Supplementary Data set 1) with no evidence of secretion. RNAs between 70 and 100 nt were also present in the secreted product (Fig. 1a) and sequencing identified full-length Y RNAs as the major component of this fraction, with two predominant classes of structure identified (Fig. 1c and Supplementary Fig. 2).

**Figure 2:**
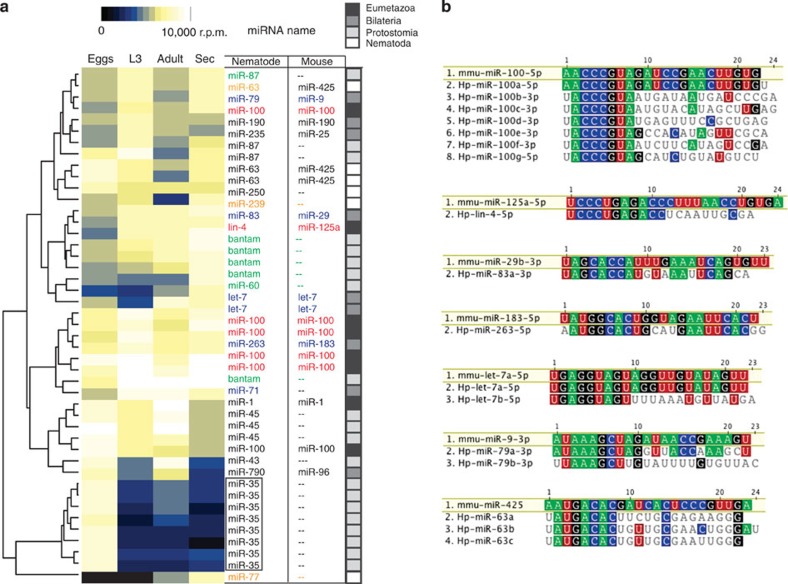
Many secreted nematode miRNAs have identical seed sites to mouse miRNAs. (**a**) Temporal expression of highly abundant miRNAs (>10,000 reads per million in at least one of the libraries) across the life stages. Nematode and mouse names are listed according to identical seed sites and miRNAs of high abundance in the secretion product are coloured according to their conservation level^18^: Eumetazoa (red), Bilateria (blue), Protostomia (green), Nematoda (orange). (**b**) Sequence alignment of abundant secreted parasite miRNAs that contain identical seed sites between mouse and *H. polygyrus*; all families shown are of common ancestry^18^ apart from miR-425/63.

A total of 263 ‘high confidence’ miRNAs were classified from the combined libraries based on representation of reads from both 5p and 3p arms of the hairpin and/or homology with known miRNAs in other nematodes (Supplementary Data set 1). Each of these matches DNA sequences in the *H. polygyrus* whole genome currently under assembly. Many also show stage-specific expression patterns: for example, miR-35 family members are exclusively expressed in the egg library, consistent with their functions in embryogenesis^17^ (Fig. 2a, boxed). The secretory products are dominated by miRNAs with identical seed sites to mouse miRNAs, many of which are ancient: six are shared among Eumetazoa (lin-4/miR-125 and five miR-100 family members, Fig. 2a, red) and five among Bilatera (miR-79/miR-9, miR-83/miR-29, miR-263/miR-183 and two let-7 family members, Fig. 2a, blue). In addition, five bantam family members dominate the secretory products along with miR-87 and miR-60, which also are shared among Protostomia (Fig. 2a, green) and three miRNAs that evolved in the nematode lineage: miR-63, miR-239 and miR-77 (Fig. 2a, orange). miR-63 shares an identical seed site to mammalian miR-425, although it is not of common ancestry^18^, Fig. 2b. On the basis of their sequences, many of the secreted parasite miRNAs could therefore hijack existing mouse miRNA target networks if taken up by host cells.

### Nematode vesicles are associated with secreted RNA

**Figure 3:**
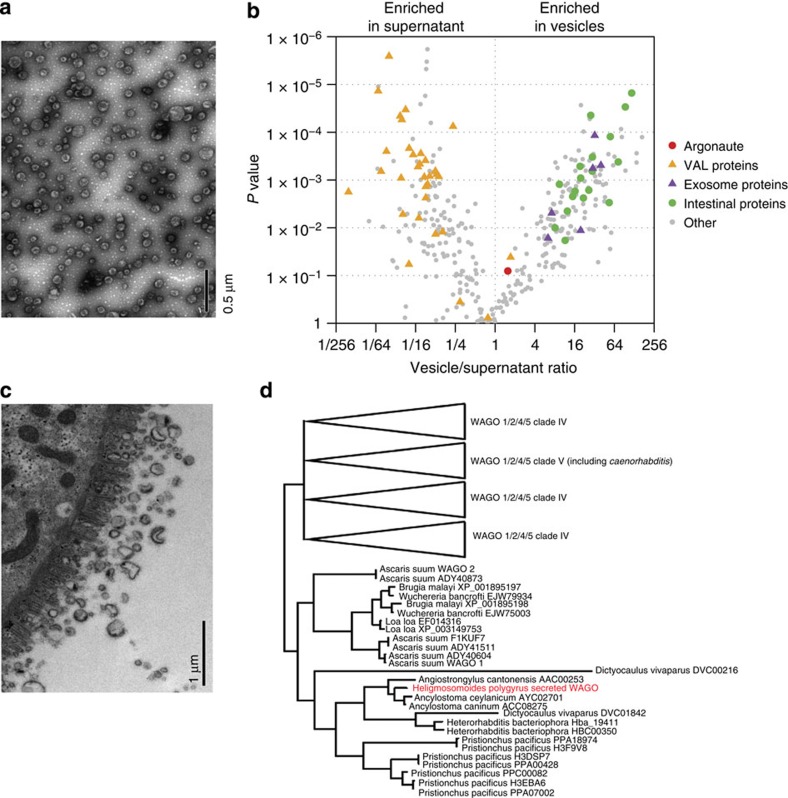
*H. polygyrus* secretes exosomes of intestinal origin that contain a WAGO protein. (**a**) TEM of purified ultracentrifugation pellet (100 μg ml^−1^ total protein) from *H. polygyrus* secretion product, scale indicates 0.5 μm. (**b**) Scatter plot of proteins enriched in ultracentrifugation pellet or supernatant based on LC-MS/MS, *n*=3, using *P*<0.05 (one-way ANOVA) and FC >1.5 as cutoffs. Noted in the legend are homologues of intestinal nematode proteins (green), mammalian exosome proteins (purple), Venom Allergen-Like (VAL) proteins (orange) and an Argonaute protein (red). (**c**) TEM of adult worm intestine noting vesicles of comparable size to exosomes, scale indicates 1.0 μm. (**d**) Phylogenetic relationship of the secreted Argonaute protein identified in *H. polygyrus* secretion product in relation to other nematode Argonautes. The analysis was performed on the same data set described in ref. 28 with the addition of the *H. polygyrus-*secreted argonaute sequence, using the same method (Bayesian analysis using MrBayes v3.2).

In mammalian systems, miRNAs have been found in body fluids in association with specific proteins or in extracellular vesicles^8^. To determine whether these RNAs could be present in vesicles, the *H. polygyrus* secretory products were ultracentrifuged and quantitative reverse transcription–PCR (qRT)–PCR used to measure miRNA levels in the pellet and supernatant, revealing the majority to be present in the pellet (Supplementary Fig. 3). Transmission electron microscopy (TEM) identified vesicle-like structures between 50 and 100 nm in diameter in the pelleted material (Fig. 3a). Label-free quantification of proteins in the vesicles and supernatant by liquid chromatography-electrospray tandem mass spectrometry LC-MS/MS identified 362 proteins, of which 139 were specifically enriched in the vesicle fraction (*P*<0.05, Fig. 3b, Supplementary Data set 2) including homologues of mammalian proteins present in exosomes: heat shock proteins, Rab proteins, tetraspanins^19^ and Alix, which is associated with exosome biogenesis^14^,^15^ (Table 1). The venom allergen-like proteins (members of the CAP superfamily, Pfam00188), which were previously identified as the dominant proteins in the *H. polygyrus* secretory products^20^, are almost exclusively in the supernatant fraction (Fig. 3b, orange), further demonstrating specificity in the molecular composition of the vesicles and possibly indicating distinct routes of secretion. On this note, nematode intestinal proteins are enriched in the vesicle fraction (Fig. 3b, green and Supplementary Data set 2) and, consistent with an intestinal origin of the nematode exosomes, vesicles of similar size are observed in the intestinal tissue of adult *H. polygyrus* analysed immediately *ex vivo* (Fig. 3c). One Argonaute protein was identified in both vesicle and supernatant fractions (Fig. 3b, red) that belongs to the clade of Worm-specific Agos (WAGO). Phylogenetic analysis suggests that homologues to this WAGO are present in many parasitic nematodes but may have been lost in *Caenorhabditis* (Fig. 3d).

### Nematode RNAs are protected from degradation by exosomes

**Figure 4:**
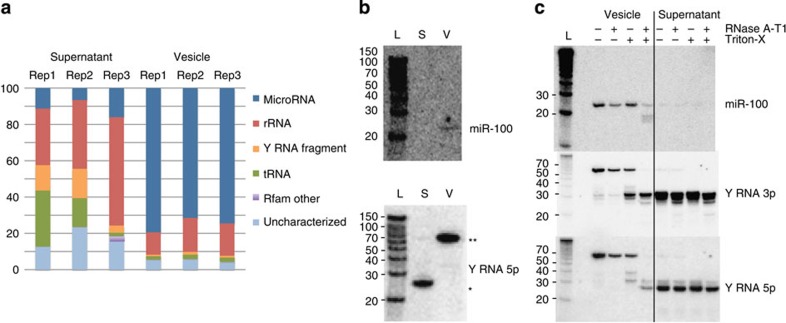
Secreted miRNAs are protected from degradation through encapsulation within exosomes. (**a**) Classification of *H. polygyrus* small RNAs in the secretion product following ultracentrifugation. (**b**) Northern blot analysis of RNA extracted from ultracentrifuge pellet or supernatant (from equivalent 10 μg protein) using probes complementary to *H. polygyrus* miR-100 or the 5′ arm of nematode Y RNA; * indicates the processed Y RNA and ** indicates the full length Y RNA. (**c**) Northern blot of RNA extracted from the pelleted secretion product following RNase treatment (0.5 Unit RNace-IT, 1 h at 37 °C) in the presence or absence of 0.05% Triton-X-100.

To determine which RNAs identified in the total secretion product (Fig. 1) are specifically associated with vesicles, small RNA sequencing of replicate vesicle and nonvesicle (supernatant) fractions of the secretion product was carried out. Results from three biological replicates demonstrate that the parasite miRNAs are enriched in the vesicle fractions (75% of reads compared with 10% in supernatant, which is dominated instead by rRNA and Y RNA fragments, Fig. 4a). This analysis also identified three mouse miRNA homologues: miR-193, miR-10 and miR-200, within the top five most abundant secreted miRNAs. These were ranked much lower in the initial Illumina analysis (Supplementary Table 1) likely because of the sequencing bias of the different kits and platforms^21^, underscoring the importance of comparing both approaches. Overall the three replicates showed the same profile of miRNAs in each vesicle sample (Supplementary Table 2) and neither vesicle nor supernatant contained intact large ribosomal RNA (Supplementary Fig. 4). Northern blot analysis confirmed the specificity of small RNA biotypes in vesicles versus supernatant, showing miR-100 to be exclusively present in the vesicles and the Y RNA fragment to be exclusively present in the supernatant (Fig. 4b). Notably, on the same blot the full-length Y RNA was detected in the vesicles and both the miRNA and full-length Y RNA were largely resistant to degradation by RNases in untreated samples but became susceptible in the presence of Triton-X-100 (Fig. 4c). Together, these results demonstrate that mature miRNAs and full-length Y RNAs are secreted by a parasitic nematode and are protected through encapsulation within vesicles of intestinal origin that share similar size and protein composition to mammalian exosomes.

### *H. polygyrus* exosomes suppress innate immunity *in vivo*

**Figure 5:**
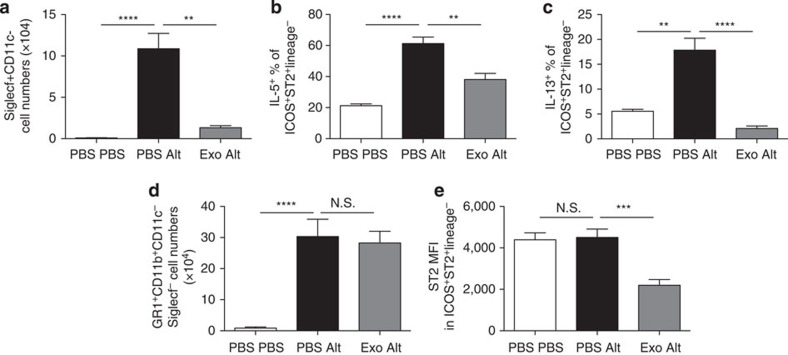
*H. polygyrus* exosomes suppress a Type 2 innate immune response *in vivo.* *H. polygyrus* exosomes (10 μg) were administered intranasally to BALB/c mice 24 h before administration of 50 μg *Alternaria* extract and a further 5 μg exosomes, or controls that received PBS. (**a**) Siglecf^+^CD11c^−^ eosinophils in the bronchoalveolar lavage; (**b**) IL-5 and (**c**) IL-13 expression in PMA/ionomycin-stimulated lineage-negative, ICOS^+^ST2^+^ group 2 innate lymphoid cells in digested lung tissue were measured 24 h after *Alternaria* extract administration; (**d**) Gr1+CD11b+ neutrophils in the same lavage samples; (**e**) the mean fluorescence intensity (MFI) of ST2 (IL33R) staining in ILCs from each group of mice. Data are representative of two independent experiments, *n*=4–6 per group; error bars are mean±s.e.m. Data analysed by ANOVA and Tukey’s post test, ****P<0.0001, ***P<0.001, **P<0.01, *P<0.05.

Helminths are well known to suppress pathogenic immune responses in both the gastrointestinal tract and airways^5^. To examine the functionality of the parasite-derived exosomes *in vivo*, they were administered intranasally in combination with extracts of the allergenic fungus *Alternaria*, which induces rapid IL-33 release as part of the Type 2 Th2-like innate immune response that leads to lung eosinophilia^22^. Pre-treatment with parasite-derived exosomes before *Alternaria* extract administration led to a sharp reduction in bronchoalveolar lavage eosinophilia (Fig. 5a), and suppressed expression of the Type 2 cytokines interleukin (IL)-5 and IL-13 by innate lymphoid cells (ILCs; Fig. 5b,c). Neutrophilia, which does not depend on Type 2 cytokines, was undiminished by exosome administration (Fig. 5d). Intriguingly, the overall expression of the IL-33 receptor (also known as ST2) was also suppressed in recipients of exosomes (Fig. 5e).

### Internalization of nematode exosomes and RNAs by mouse cells

**Figure 6:**
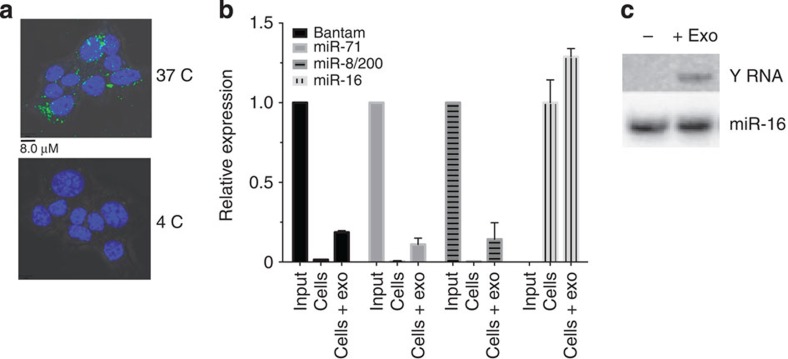
*H. polygyrus* exosomes and RNAs are internalized by mouse cells. (**a**) Confocal analysis of murine epithelial cells incubated for 1 h with PKH67-labelled *H. polygyrus* exosomes at 37 and 4 °C, scale indicates 8.0 μM. (**b**) Relative expression of parasite-derived miRNAs in murine epithelial cells at 20 h post incubation with 5 μg *H. polygyrus* exosomes following PBS washes. Signal observed in untreated host cells represents the background detection of the probe; for parasite-derived miRNA, the data are normalized to the input detection level of miRNAs in 5 μg of exosomes, whereas miR-16 levels in exosome-treated cells are normalized to untreated cells. (**c**) Northern blot analysis of RNA extracted from murine epithelial cells following 20 h incubation with *H. polygyrus* exosomes (5 μg total protein) compared with untreated cells following PBS washes, using a probe against the loop of the nematode Y RNA or mouse miR-16.

To determine whether the nematode-derived exosomes can enter mammalian cells, uptake was examined in mouse small intestinal epithelial cells, a cell type that is naturally in direct contact with *H. polygrus in vivo*. Exosomes were labelled with the lipid dye PKH67 and incubated with MODE-K cells *in vitro*. Uptake was analysed by fluorescence-activated cell sorting (FACS) and confocal microscopy. Over 60% of the cells were PKH67-positive after 1 h of incubation with *H. polygyrus* vesicles compared with 1.5% when incubated with background dye (Supplementary Fig. 5). These results are unlikely to be due to nonspecific association with the cell membrane as treatment with trypsin did not eliminate the signal (Supplementary Fig. 5). Confocal analysis confirmed uptake to the cytoplasm and demonstrates that this requires physiological temperature (Fig. 6a). qRT–PCR analysis of the treated cells detects the parasite-specific miRNAs in cells after 20 h of incubation, with no change in the endogenous miR-16 (Fig. 6b). The full-length parasite-derived Y RNA could also be detected by northern blot analysis in cells which were treated directly with exosomes followed by washing (Fig. 6c).

### Regulation of mouse genes by nematode exosomes

**Figure 7:**
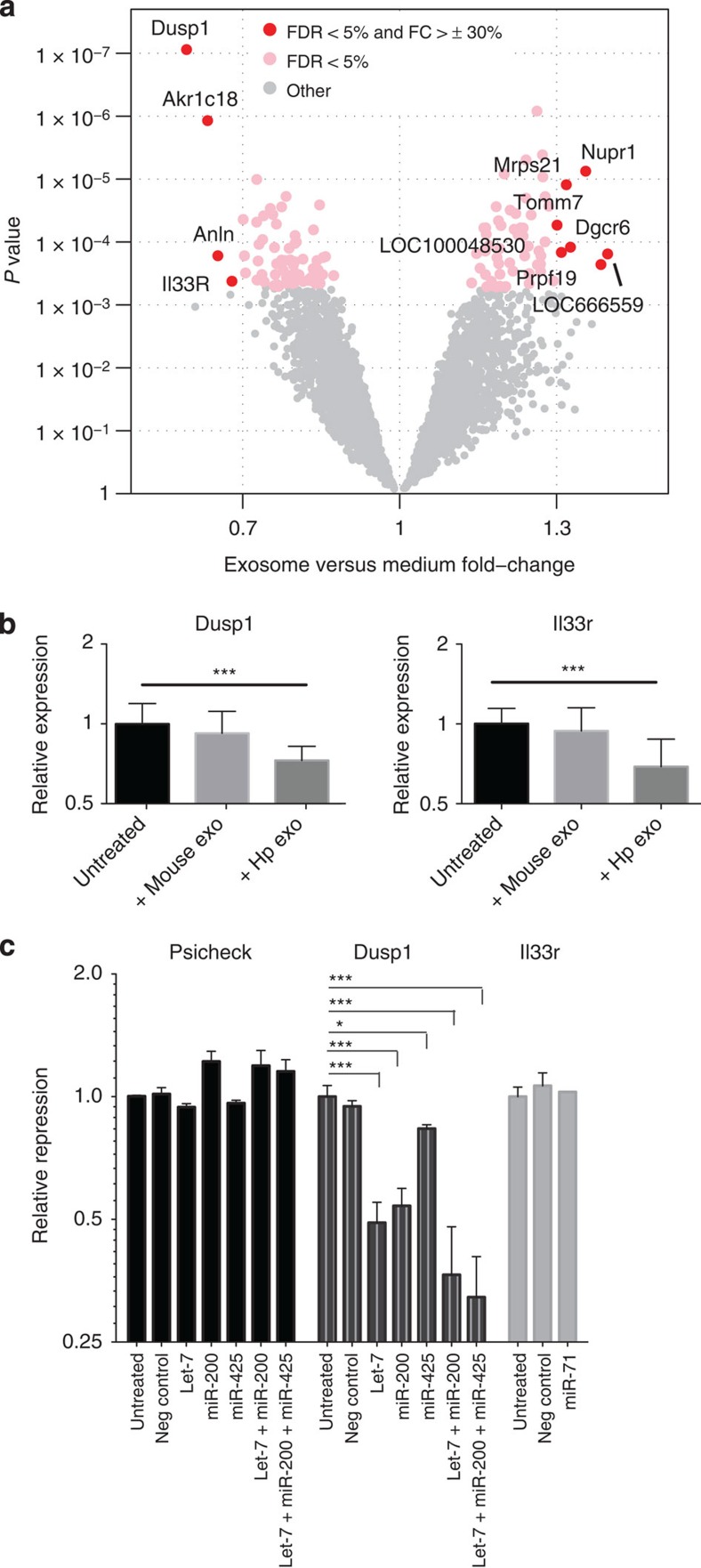
Mouse *Il33r* and *Dusp1* are suppressed by *H. polygyrus* exosomes and the secreted miRNA repress target sites in *Dusp1.* (**a**) Volcano plot of mouse genes up- or downregulated upon incubation with *H. polygyrus*-derived exosomes; red=FDR *P*<0.05 and FC>30%. (**b**) Levels of *Dusp1* and *Il1rl1* in mouse epithelial cells (5 × 10^4^) following 48 h treatment with 5 μg *H. polygyrus* exosomes or MODE-K-derived exosomes, *n*=8, error bars are mean±s.e.m. Data analysed by ANOVA and Tukey’s post test, *P<0.05, **P<0.01, ***P<0.005, ****P<0.001. (**c**) Repression of Psicheck reporter vector containing *Dusp1* or *Il1rl1* 3′UTRs fused to Renilla luciferase by co-transfection with individual or pooled synthetic *H. polygyrus* miRNAs (50 nM), data represent renilla/luciferase ratios, normalized to the values obtained for untreated samples; *n*=3, ***P<0.005, *P<0.05.

To determine the function of these vesicles in mouse cells, gene expression analyses were carried out on MODE-K cells following incubation with *H. polygyrus* exosomes. A total of 128 genes were differentially expressed upon treatment (false discovery rate (FDR) *P*<0.05). Relatively subtle changes in gene expression were observed (Fig. 7a); however, the most strongly downregulated gene was *Dusp1* (also known as *MKP-1* in human), a key regulator of MAPK signalling associated with dampening the type 1 pro-inflammatory reaction to Toll like receptor (TLR) ligands. Another gene significantly downregulated by exosomes is *Il1rl1* (also known as *IL33R* in human and so referred to here as *Il33r*), the ligand-specific subunit of the receptor for IL-33, a key alarmin cytokine required for protection against multicellular parasites, which is produced by innate cells to drive early type 2 immune responsiveness^23^ and is suppressed by the exosomes in ILCs *in vivo* (Fig. 5e). The effects of the exosomes on *Dusp1* and *Il33r* were validated by RT–qPCR and are unlikely to reflect a nonspecific response to vesicle uptake as exosomes derived from mouse intestinal cells showed similar uptake but did not alter *Dusp1* and *Il33r* levels (Fig. 7b and Supplementary Fig. 5).

There are a number of potential mechanisms that could mediate the decrease in *Dusp1* and *Il33r* levels. The 3′untranslated region (UTR) of *Dusp1* is highly conserved and contains 7mer binding sites for the parasite homologues of mouse miR-200 (aka miR-8) and let-7 as well as a 6mer site for miR-425 (aka miR-63) in between these sites (Supplementary Fig. 6). We therefore examined whether the parasite miRNAs could suppress translation of a reporter vector containing the 3′-UTR of *Dusp1* fused to luciferase. Synthetic parasite miRNAs were transfected into MODE-K cells, resulting in 1.2- to 2.0-fold reduction in luciferase levels for the *Dusp1* reporter but not control (Fig. 7c). Notably, transfection of a cocktail of three of the miRNAs (at the same total RNA concentration) resulted in an increased reduction in luciferase activity (3.1-fold). This is consistent with enhanced repressive effects of miRNA sites in close proximity and suggests that secreted parasite miRNAs could work in cooperation to exert maximal effects on host genes. In contrast, the 3′-UTR of the IL33R-encoding gene *Il1lr1* is not conserved and, although binding sites for some of the secreted miRNAs were identified, we did not observe repression of a *Il1rl1* 3′-UTR reporter by transfection of miR-71, which contained two 7mer sites (Fig. 7c and Supplementary Fig. 6).

### Circulating nematode miRNAs in serum

**Figure 8:**
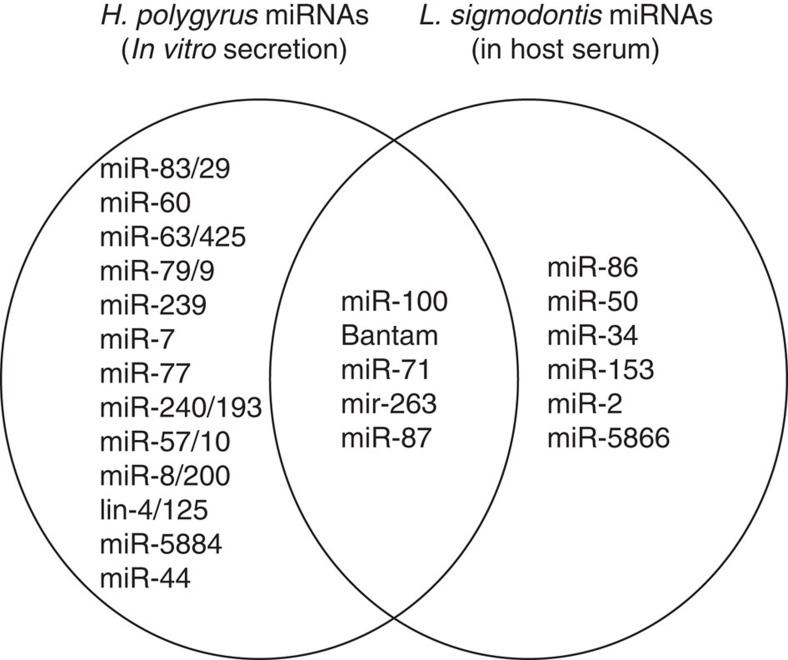
Venn Diagram of overlap in miRNAs identified in *H. polygyrus* secretion product or serum of mice infected with *L. sigmodontis.* The *H. polygyrus* miRNAs for comparison are taken from Supplementary Table 1 (top 20 most abundant in at least one platform). The miRNAs that are perfectly conserved between nematodes and mice are excluded, since the origin in serum cannot be deduced.

To establish whether the secreted nematode miRNAs naturally circulate in host tissues *in vivo*, we examined serum from mice infected with *H. polygyrus* (which resides in the gut lumen) or the filarial nematode *L. sigmodontis* (which resides in the pleural cavity). No *H. polygyrus* miRNAs were detected in the serum; however, a total of 1,188 reads mapped perfectly and unambiguously to the *L. sigmodontis* draft genome and 761 of these derived from 16 nematode miRNAs (Table 2 and Supplementary Table 3). Although we cannot rule out the possibility that some of the miRNAs in serum could derive from dying worms, the most abundant miRNAs detected are homologues of those found in *H. polygyrus* exosomes, including miR-100, bantam, miR-71 and miR-263 (Table 2, Fig. 8). These data confirm the *in vivo* secretion of parasite miRNAs and are consistent with the idea that exosomes and associated RNAs operate locally in the host’s body such that their detection in body fluids will be dictated by the life stage and localization of the parasite in the host.

## Discussion

In summary, we have shown that nematode parasite-derived miRNAs and Y RNAs are transported into mammalian host cells via exosomes that regulate host genes associated with immunity and inflammation and suppress an innate Type 2 response *in vivo*. Extracellular vesicles are emerging as a central mechanism for cell-to-cell signalling within mammalian systems, and our report of their secretion by a nematode species is within the setting of vesicle secretion by an increasingly diverse range of pathogens^24^,^25^,^26^. We have demonstrated for the first time that nematode-derived RNAs are a key component within exosomes that can be transferred to host cells. Nematodes are ubiquitous pathogens of both plants and animals and we anticipate that RNA secretion is a conserved phenomenon, supported by the fact that we detect miRNAs from the filarial nematode *L. sigmodontis* in host tissue, consistent with a recent report^12^. In fact, RNA secretion may be a ubiquitous feature across a range of parasites; an initial report suggests that miRNAs are also associated with vesicles in the trematode *Dicrocoelium dendriticum*^27^.

Given that many of the nematode miRNAs are homologues to mouse miRNAs, it is tempting to speculate that these could tap into existing miRNA regulatory networks in host cells. In support of this, we show with a reporter assay that three of the secreted nematode miRNAs that have identical seed sites to mouse miRNAs can together downregulate DUSP1 through conserved sites in its 3′UTR. Many questions remain, however, regarding the mechanism by which the nematode miRNAs can operate in host cells. The exosome is a functional ensemble and immune suppression is likely to require a combination of protein and miRNAs for fusion and gene regulation. It will be challenging, therefore, to pin point the individual contributions of each. For example, a nematode Ago protein is secreted with the miRNAs that may be required for functionality. This Ago belongs to the WAGO clade of Agos that evolved in the nematode lineage. The WAGOs mediate diverse RNA interference mechanisms in nematodes and can operate at epigenetic, transcriptional and post-transcriptional levels^28^; it is intriguing to now consider how these possibilities could extend to their hosts.

An exciting finding in this study is the fact that the exosomes can suppress an innate Type 2 response *in vivo*, identifying vesicles as another class of immunomodulator used by the parasite and opening the door to further exploitation of exosomes in a therapeutic context. Our previous work has shown that *H. polygyrus*-secreted material suppresses IL-33 release and it is likely that a combination of soluble proteins and exosomes together suppress this important pathway^22^. From analyses *in vitro* we identify *Il33r* and *Dusp1* as host genes directly suppressed by the exosomes. Although DUSP1 has been broadly viewed as an attenuator of immune activation, it is known to preferentially downregulate IL-6 that has recently been shown to promote susceptibility to *H. polygyrus*^29^, while upregulating IL-10, which acts as a broadly immunosuppressive cytokine^30^. Hence, parasite survival is likely to be favoured by reduced DUSP1 levels. Further, DUSP1/MKP-1 dampens the acute inflammatory response to lipopolysaccharide, promoting macrophage arginase expression over nitric oxide synthase^31^. Hence, parasite repression of DUSP1 could block the induction of arginase, a known mediator of killing of *H. polygyrus* in the mouse^32^. These possibilities are now being investigated in our laboratories. Our reporter assays suggest that Dusp1 could be directly targeted by the parasite miRNAs; however, we do not observe repression of Il33r when transfecting the parasite miRNA, miR-71, that is predicted to target its 3′UTR. It may be that additional parasite-derived RNAs or proteins could regulate *Il33r* expression, or that the effect operates indirectly through a separate target gene. For example, reduced expression of *Dusp1* or other regulators of MAPK signalling could result in GATA-2 phosphorylation, which might inhibit its ability to promote *Il33r* transcription^33^,^34^.

Finally, our work has revealed not only secreted miRNAs that are packaged in exosomes but also full-length Y RNAs that are transferred to host cells at an abundance level detectable by northern blot analysis. Y RNAs are not known to function in gene silencing but were recently shown to be packaged into exosomes secreted from dendritic cells^35^ and play roles in RNA quality control and DNA replication in humans^36^. Further work is required to understand whether and how each of these classes of secreted parasite RNA can contribute to the capacity of this parasite to manipulate its environment within the host.

## Methods

### Purification of vesicles from secretion product

For collection of *H. polygyrus* secretion product, CF1 mice are infected with infective-stage larvae by gavage and adult parasites collected from the small intestine 14 days post infection. The worms are maintained in serum-free media *in vitro* as described elsewhere^37^; secretion product is collected every 3 days for a maximum of 3 weeks (samples used here were from the first week of collection) and purified as follows: eggs are removed by spinning at 400 *g* before filtering of the secretion through 0.2-μm filter (Millipore). Filtered media is then processed following a modified protocol from that described in ref. 38, by being spun at 100,000 *g* for 2 h in polyallomer tubes at 4 °C in a SW40 rotor (Beckman Coulter). Pelleted material is washed two times in filtered PBS at 100,000 *g* for 2 h. The supernatant is concentrated using Vivaspin 6 5000 MWCO tubes (Fisher) at 5,000 *g* and washed two times with PBS.

### Small RNA library preparation and analysis

For the analysis of small RNAs in the life stages and total secretion products, total RNA was size-selected on 15% denaturing PAGE and libraries prepared from the 18 to 30 nt fraction using Illumina Small RNA preparation kit version 1.5 and sequenced on an Illumina GAIIX instrument in Edinburgh Genomics ( http://genomics.ed.ac.uk/). To identify larger RNAs in the secretion product, separate libraries were also prepared for RNA size selected between 60 and 100 nt and sequenced in parallel. For analysis of vesicle and nonvesicle fractions, small RNA libraries were prepared using the TruSeq kit and sequenced on MiSeq platforms, without prior size fractionation of the RNA. All libraries were analysed by first clipping the 3′ sRNA adapter using cutadapt, searching for at least a six-base match to the adapter sequence. For analysis of small RNAs only reads that contained the adapter were 16–40 nt in length and were present at more than two copies were retained for further analysis. For analysis of RNAs >60 nt in the secretion product, sequences present at >100 reads in the library (out of 490,614 reads sequenced) were aligned in Clustalw and manually inspected for sbRNA (Y RNA) content in terms of secondary structure and location of a UUAUC motif in the terminal loop as described in^16^ (Fig. 1c and Supplementary Fig. 2). The Y fragments in the small RNA libraries (<40 nt) were then identified based on criteria that they aligned to these full-length Y RNAs.

The draft genome assembly for *H. polygyrus* was created with the CLC *de novo* assembler using two lanes of Illumina GAII data with 50 bp paired-end and 100 bp paired-end reads from Edinburgh Genomics ( http://genomics/ed.ac.uk/); the raw and assembled data are available at http://heligmosomoides.nematod.es/. This version was used to map the sequences around small RNA reads to identify structures consistent with miRNA precursors (according to prediction programmes detailed below). Reads matching the genome were aligned to a set of RNA sequences consisting of known *H. polygyrus* 18S, 28S and 5.8S rRNA sequences (Genbank AJ920355.1, AM039747.1 and DQ408618.1:527-678), 5S rRNA from a closely related species (*Trichostrongylus colubriformis*, Genbank U32119.1) and Rfam sequences (version 10, obtained from ftp://ftp.sanger.ac.uk/pub/databases/Rfam/10.0/Rfam.fasta.gz). The best hit with at most two edits was used to classify the reads. Any reads that matched an rRNA or non-microRNA Rfam family were filtered before miRNA analysis. The analysis of piRNAs was carried out with reads that did not match known Rfam classes or miRNAs; initial identification was based on the presence of a ‘GUUUCA’ between 35 and 65 nt upstream of the 5′ RNA alignment start site^39^. Inspection of the distribution plot identified the region 42–45 nt upstream of the 5′ RNA alignment start site as being the key area for subsequent analyses (Supplementary Fig. 1).

Two miRNA prediction programmes were used to identify miRNAs in the data sets: miRDeep2 (ref. 40) and mireap ( http://sourceforge.net/projects/mireap/). Both programmes use miRNA biogenesis to model the expected alignment of sRNA reads to a potential miRNA precursor. For miRDeep2, the following default settings were used: (a) requirement that reads match the genome perfectly, (b) removal of reads that match to more than five places in the genome and (c) cutoff -v 1, (d) the ‘-s option’ was employed, using all mature sequences from mirbase (version 19). The default settings of minimum free energy (<−20 kcal mol^−1^) and read length (18–30) were employed. In both programmes, precursor predictions with fewer than 10 reads were discarded. Where multiple precursor loci predicted identical mature miRNAs, only the precursor with the largest number of matching reads was reported.

### pCp end labelling and northern blot

For 3′ end-labelling, total RNA was extracted from the life stages and secretion product using the miRNAeasy kit (Qiagen): 1 μg total RNA was used from life stages and RNA extracted from a volume of secretion product equating to 15 μg protein (the total RNA concentration was too low to detect by nanodrop or qubit). The 3′-end labelling was carried out at 4 °C overnight in 10 μl using RNA ligase I (NEB) according to the manufacturer’s instructions with 3,000 Ci mmol^−1 32^P PcP (Perkin Elmer). Reactions were quenched by the addition of 2 × loading buffer (8 M urea, 0.5% TBE) and 4 μl run on an 18% PAGE at 350 V for 8 h, which was then visualized by phosphorimaging using a Typhoon Scanner (GE Healthcare). For northern blot analysis, total RNA was extracted from volumes of vesicle and nonvesicle fractions that contained equivalent protein (10 μg) and then separated by denaturing 15% PAGE, transferred to Hybond-N+ membrane (GE Healthcare) and chemically crosslinked as described previously^41^. Blots were prehybridized in PerfectHyb (Sigma) for 1 h at 42 °C before overnight incubation with DNA probes (Invitrogen) that were perfectly complementary to the miRNA or Y RNA: miR-100: 5′-ACACAAGTTCGGATCTACGGGTT-3′, YRNA-5P: 5′-ACCCTACGACTCCGGACCAAGCGCG-3′, YRNA-3P: 5p-GCGCCGGTCGAGCTTTTGTCGAAGGGAAT-3p, Y RNA-loop: 5p-AAGGGAATTCGAGACATTGTTGATAAC-3p. The probes were labelled with T4 PNK (NEB) and 6,000 Ci mmol^−1 32^P ATP (Perkin Elmer) according to the manufacturers’ protocols.

### miRNA RT–qPCR

Analysis of miRNA levels in ultracentrifugation fractions was carried out using the miScript system (Qiagen) with unmodified DNA probes identical to the full-length parasite miRNA (Life Sciences): miR-100: 5′-AACCCGTAGATCCGAACTTGTGT-3′, miR-71: 5′-TGAAAGACATGGGTAGTGAGAC-3′, let-7: 5′-TGAGGTAGTAGGTTGTATAGTT-3′ and miR-60: 5′-TATTATGCACATTTTCTGGTTCA-3′. For analysis of parasite-derived miRNA levels in host cells, qRT–PCR was carried out using the miRCURY LNA microRNA PCR system (Exiqon) and LNA probes were custom-designed by Exiqon to minimize cross hybridization with mouse sequences, and efficiency of probes was measured between 90 and 100% (data not shown). Analysis of mouse gene expression levels was carried out using the Sybr green I master mix (Roche), with the following primers: gapdh_F: 5′-CATGGCCTTCCGTGTTCCTA-3′, gapdh_R: 5′-GCGGCACGTCAGATCCA-3′ Dusp1_F: 5′-GTGCCTGACAGTGCAGAATC-3′, Dusp1_R: 5′-CACTGCCCAGGTACAGGAAG-3′, Il33R_F: 5′-AGACCTGTTACCTGGGCAAG-3′, Il33R_R: 5′-CACCTGTCTTCTGCTATTCTGG-3′. Data were collected on a Light Cycler 480 System (Roche) following temperature profiles recommended by each manufacturer. The delta *C*_t_ method was used for quantification as described in ref. 11 using GAPDH as the normalizer. Data were analysed using one-way analysis of variance (ANOVA) followed by Tukey’s post test and variance within groups assessed by Brown Forsythe test.

### LC-MS/MS

Five micrograms of total protein from the secretion product ultracentrifuge pellet or supernatant were loaded on a 12% Tris-Bis NuPAGE gel (Invitrogen) and electrophoresis carried out for 5 min before in-gel digestion as described in ref. 42. Capillary-HPLC-MS/MS analysis was performed using an online system consisting of a micropump (1,200 binary HPLC system, Agilent, UK) coupled to a hybrid LTQ-Orbitrap XL instrument (ThermoFisher, UK). Data were searched using MASCOT Versions 2.4 (Matrix Science Ltd, UK) against an in-house *H. polygyrus* transcriptome assembly of 454 sequences^43^ using a maximum missed-cut value of 2. Variable methionine oxidation and fixed cysteine carbamidomethylation were used in all searches; precursor mass tolerance was set to 7 p.p.m. and MS/MS tolerance to 0.4 a.m.u. The significance threshold (*p*) was set below 0.05 (MudPIT scoring). A peptide Mascot score threshold of 20 was used in the final analysis, which corresponds to a global FDR of 4.6% using a decoy database search. LC-MS label-free quantitation was performed using Progenesis (Nonlinear Dynamics, UK) as described elsewhere^42^ where the total number of Features (that is, intensity signal at a given retention time and m/z) was reduced to MS/MS peaks with the charge of 2, 3 or 4+ and we only kept the five most intense MS/MS spectra per ‘Feature’. The subset of multicharged ions (2+, 3+ and 4+) was extracted from each LC-MS run. For a specific protein, the associated unique peptide ions were summed to generate an abundance value that was transformed using an ArcSinH function required for the calculation of the *P* value. A total of 362 proteins were identified in either the supernatant or pellet based on requirement of at least two peptides present; of these, 122 were enriched in the supernatant and 139 in the pellet, while the remaining 101 did not show statistically significant enrichment and were detectable in both samples. The within-group means were calculated to determine the fold change and the transformed data were then used to calculate the *P* values using one-way ANOVA. Differentially expressed proteins were considered meaningful under the following conditions: detected by two or more peptides, with an absolute ratio of at least 1.5 and *P*<0.05 associated with the protein change. Classification of intestinal proteins is based on homology to proteins identified in other nematodes, described in ref. 44.

### TEM

For visualization of the vesicles, the purified ultracentrifuged pellet from *H. polygyrus* secretion product (100 μg ml^−1^ protein concentration) was fixed in 2% paraformaldehyde (PFA), deposited on Formvar-carbon-coated EM grids and treated with glutaraldehyde before treatment with uranyl oxalate and methyl cellulose as described in ref. 38. For analysis of adult *H. polygyrus* parasites, samples were washed with PBS before fixation in 2.5% glutaraldehyde solution in 0.1 M sodium cacodylate buffer overnight. Parasites were rinsed three times with 0.1 M Na cacodylate buffer, and post-fixed in 1% osmium tetroxide for 1 h. After rinsing in 0.1 M Na cacodylate buffer, they were sequentially dehydrated in a graded acetone series. Finally, samples were sequentially incubated for 30 min in an araldite:acetone solution left to evaporate overnight at 60 °C and then embedded in fresh araldite resin and polymerized at 60 °C for 48 h. Ultrathin sections, 60-nm thick, were cut from selected areas, stained in uranyl acetate and lead citrate, and then viewed in a Philips CM120 TEM. Images were taken on a Gatan Orius CCD camera.

### Flow cytometry and confocal analyses of uptake

Purified exosomes from *H. polygyrus* or MODE-K cells (measured as 5 μg of total protein) were labelled with 2 μg of PKH67 dye (Sigma) for 5 min at room temperature following the manufacturer’s protocol. The staining reaction was stopped by adding an equal amount of 1% bovine serum albumin (BSA), and exosomes were washed in PBS and pelleted by ultracentrifugation (1 h at 100,000 *g*). A probe solution was prepared with the PKH67 following the same protocol but mixed with PBS solution in the absence of exosomes. MODE-K cells^45^ were obtained from Dominique Kaiserlian (INSERM) and grown following the standard protocol in DMEM (Invitrogen) medium supplemented with 10% fetal bovine serum (Invitrogen), 1% penicillin–streptomycin (Lonza), 1% L-glutamine (Lonza), 1% non-essential amino acids/sodium pyruvate (Gibco). These were mycoplasma-free based on testing every 4 weeks. On the day of the experiment, cells were seeded in 24-well plates (1 × 10^5^ cells per well) using advanced DMEM serum-free medium (Invitrogen) supplemented with 1% L-glutamine and subsequently incubated (1 h at 37 °C) either in the presence of PKH67-labelled *H. polygyrus*-derived exosomes (5 μg of total protein) or in the presence of the probe alone. After incubation, cells were harvested, washed twice in FACS buffer (PBS 1 × , 2.5% FBS, 0.1% BSA, 0.05% NaN_3_) and finally resuspended in 500 μl of the same buffer. A subset of the samples were then incubated with 50 ul of 0.25% Trypsin/EDTA (Gibco) for 5 min before analysis (indicated in Supplementary Fig. 5). Samples were analysed using the BD FACS Canto II flow cytometer (BD Bioscience). Data files were acquired from the cytometer, with 5,000 events collected for each tube and the data analysis was performed using the FlowJo software (Tree Star Inc.). For confocal analyses, MODE-K cells were seeded on round microscope cover glasses in 24-well plates (2.5 × 10^4^ cells per well) in media described above. Cells were allowed to attach on to the coverslips overnight and the following day shifted to advanced DMEM medium supplemented with 1% L-glutamine. Cells were incubated (1 h at 37 or 4 °C) either in the presence of labelled exosomes or probe only. After incubation, medium was aspirated, cells were washed twice in PBS and fixed with 4% PFA, with residual PFA quenched with 50 mM glycine. Slide coverslips were washed extensively in PBS, and nuclei were stained with 4',6-diamidino-2-phenylindole-supplemented ProLong Fade Gold (Invitrogen) mounting media. Samples were examined on the Leica SP5 II (Leica Microsystems, lasers exciting at 405 and 488, × 63 objective) using the LAS AP software (Leica). Images were analysed using the Volocity software (Improvision).

### Microarray analysis

MODE-K cells were grown in DMEM media as described above and seeded into 24-well plates at 20,000 cells per well. The following day, cells were incubated with *H. polygyrus*-derived exosomes (5 μg total protein per well) for 20 h before washing twice with PBS and total RNA extracted. RNA was prepared for microarray analysis using the Illumina TotalPrep RNA Amplification kit and run on MouseWG-6 v2.0 (Illumina) at the Wellcome Trust Clinical Research Facility (University of Edinburgh). The raw SampleProbeProfile file was processed within R, using ‘lumi’ and ‘lumiMouseAll.db’ Bioconductor packages^46^,^47^,^48^. Quality control was performed using Multi-Dimensional Scaling, and one of the control samples that behaved as an outlier was removed. Raw expression values were processed with the Variance Stabilizing Transformation and the Robust Spline Normalization^49^. An InterQuartile Range was calculated across all samples for each probe, and used to select the most variable probe of those that mapped to the same transcript. Probes without a gene or transcript annotation were excluded, leaving a total of 30,708 nonredundant annotated probes. Differential expression was performed using the ‘limma’ package^50^, fitting a linear model for each probe and using an empirical Bayes method to obtain moderated t-statistics. In order to reduce the multiple-test problem and focus on the most interesting genes, ‘present’ probes with an Illumina detection *P* value <0.05 in at least three samples were selected, leaving 12,276. The Benjamini and Hochberg method was used to calculate FDRs^51^.

### *In vivo* analysis of exosome function in *Alternaria* model

BALB/c mice were bred in-house at the University of Edinburgh and accommodated according to Home Office regulations. Female mice were used when they were 6–10 weeks old. For all experiments presented in this study, the sample size was large enough to measure the effect size. No randomization and no blinding were performed in this study. *H. polygyrus* exosomes (10 μg) were administered intranasally (under isoflurane sedation) in 50 μl PBS, or 50 μl PBS alone to controls, 24 h before intranasal administration of 50 μg *Alternaria* extract with a further 5 μg of exosomes. Mice were killed 24 h after *Alternaria* administration, and bronchoalveolar lavage and lung cell suspensions stained for flow cytometry as described previously^22^. Briefly, cells were counted, then surface stained for Siglecf+CD11c− (eosinophils) or stimulated with phorbol myristate acetate (PMA) and ionomycin for 4 h in the presence of BrefeldinA and surface stained as negative for lineage markers (CD3/CD4/CD5/CD19/CD11b/CD11c/CD19/GR1) and positive for CD45, ICOS and ST2 (ILC2s), and assessed for staining of IL-5 and IL-13. Samples were acquired on a Becton-Dickinson LSRII flow cytometer.

Data were analysed using Prism 6 (Graphpad Prism, La Jolla, CA, USA). Variance within groups was assessed by Brown Forsythe test and data were log-transformed and analysed by one-way ANOVA, with a Tukey’s multiple comparisons post test. Unless otherwise indicated, differences are not significant. ****P<0.0001, ***P<0.001, **P<0.01, *P<0.05, N.S. not significant *P*>0.05.

### Luciferase assays

The 3′UTRs of Dusp1 and Il33r were cloned behind *Renilla* luciferase in the Psicheck2 vector (Promega) at NotI and XhoI restriction sites as described in ref. 41 using the following primers: Psi-Dusp_F: 5′-CTTTACTCGAGAGGTGTGGAGTTTCACTTGC-3′, Psi-Dusp_R: 5′-CTTTAGCGGCCGCAGCTACAAACCTACACTGGC-3′, Psi-Il33r_F: 5′-CTTTACTCGAGGACTGTGTGTTGTAGCTTGG-3′, Psi-Il33r_R: 5′-CTTTAGCGGCCGCCAGAGGGAGGCTTTATAAGG-3′.

For reporter assays, 15,000 cells were reverse transfected into a 96-well plate with 0.3% lipofectamine (Invitrogen) and 50 ng of each Psicheck reporter in the absence or presence of 50 nM synthetic miRNA mimic (Thermofisher). Luciferase measurements were carried out at 48 h post transfection, using the Dual Glo Luciferase assay system (Promega) and Luminensence measured on a Varioskan plate reader (Thermofisher). Data shown in Fig. 7b represent *n*=3 replicates (separate transfection experiments of the MODE-K cell line) measured in parallel to control for consistent Renilla and Luciferase ratios using the same kit; data were analysed by one-way ANOVA, with a Dunnett’s post test, ****P<0.0001, ***P<0.001, **P<0.01, *P<0.05.

### MicroRNA target prediction

A custom Perl script was used to identify seed-matching sites for the *H. polygyrus* miRNAs identified by sRNA-Seq. All the 3′UTR sequences corresponding to probes on the microarray were scanned, and the results were passed to the TargetScan (v6.2) context scores Perl script. Targets for each miRNA were ranked by ‘Context+ Scores’. Conservation scores for relevant 3′UTRs (Dusp1 and Il33r) were obtained from the UCSC Genome Browser, using the ‘rtracklayer’ package to access the ‘phastCons60wayPlacental’ table^52^,^53^.

### Sequencing analysis of serum from infected mice

F1 mice were infected with *H. polygyrus* (400 L3 larvae introduced by oral gavage) and serum collected on day 14 post infection. The presence of adult parasites was confirmed by visual inspection of the mouse gut lumen. Six-week-old BALB/c mice were infected with *L. sigmodontis* (subcutaneous inoculation of 40 L3), and gel-separated serum (BD Microtainer) was collected by arterial exsanguination at 60 days post infection, which was confirmed by detection of adult worms in the mouse pleural cavity and microfilarie in peripheral blood. For library preparation, 200 μl of serum was extracted with the miRNAeasy kit (Qiagen) and libraries generated following the Trueseq protocol and sequenced on the Illumina Rapid HighSeq in Edinburgh Genomics. Data were processed as described above and analysed for perfect alignment to the mouse or *L. sigmodontis* genome (reads mapping to both genomes were not analysed). Reads that aligned were then categorized by matches to Rfam or prediction as miRNAs with miRdeep2, as described in ref. 11.

## Author contributions

A.H.B. designed and carried out RNAseq experiments and validation, identified vesicles in secretion, co-designed and contributed to analysis of serum and proteomic data, co-supervised functional and uptake assays and wrote the paper; G.C. co-designed and carried out functional analyses, *in vivo Alternaria* extract experiments and vesicle detection in nematodes; F.S. designed and carried out uptake and functional assays; S.K. carried out genome alignment/annotation; H.M. co-designed and performed *in vivo Alternaria* extract experiments; J.Q. prepared and co-analysed serum libraries, T.L.B. carried out LC-MS/MS and analysis; C.A.-G. analysed microarray data and target prediction; M.L. purified secretion material and supported functional studies; Y.H. generated worm and secretion samples; A.C. supported uptake and reporter assays; M.B. oversaw genome assembly and phylogenetic analyses of AGO protein; S.A.B. performed the *L. sigmodontis* infections and tissue sampling; A.I. analysed small RNAseq data; R.M.M. supplied *H. polygyrus* life stage material, contributed to analysis of proteomic data, co-designed immunological experiments and edited the manuscript.

## Additional information

**How to cite this article:** Buck, A. H. *et al.* Exosomes secreted by nematode parasites transfer small RNAs to mammalian cells and modulate innate immunity. *Nat. Commun.* 5:5488 doi: 10.1038/ncomms6488 (2014).

**Accession codes:** The sequencing and microarray data from this study have been deposited in Gene Expression Omnibus ( http://www.ncbi.nlm.nih.gov/geo/) under accession codes GSE55978 and GSE55941. The novel miRNA sequences identified in this study have been deposited in miRBase and the official naming is provided in Supplementary Data 1. The genomic information for *H. polygyrus* and *L. sigmodontis* is available at http://www.nematodes.org/genomes/heligmosomoides_polygyrus/ and http://nematodes.org/genomes/litomosoides_sigmodontis/.

## Supplementary Material

Supplementary Figures and Tables Supplementary Figures 1-6 and Supplementary Tables 1-3.

Supplementary Dataset 1 piRNAs and miRNAs identified in H. polygyrus libraries with read counts indicated for each library. For each mature miRNA sequence, seed-site identity (positions 2-7) in other organisms is indicated. Pre-miRNAs with reads mapping to both arms of the hairpin and/or which contain homology to other nematode miRNAs are defined as "high confidence", while those that do not fulfil this criteria are "low confidence".

Supplementary Dataset 2 *H. polygyrus* proteins identified in vesicle and non-vesicle fractions of the secretion product by LC-MS/MS. Identity of the most highly similar matches in the NCBI nr database using BLAST is shown as well as relative enrichment and p values (n=3). Those noted as exosome proteins, VAL proteins, Intestinal proteins and the Ago protein in Fig. 3 are indicated.

## Figures and Tables

**Table 1:** Table of nematode proteins enriched in vesicle fraction that are homologous to mouse proteins associated with exosomes.

**Name**	**Pellet/sup ratio**	***P***-**value**	**Organism**	**Blast** ***E*** **value**
Tetraspanin-11	40.0	<0.005	*A. suum*	2e−46
Hsp-70	32.2	<0.005	*D. medinensis*	0.0
Alix	30.2	0.006	*C. elegans*	1e−79
Rab-11b	19.7	0.011	*S. salar*	1e−71
Rab-5	7.2	0.005	*C. elegans*	2e−205
Hsp-90	6.3	0.016	*H. contortus*	0.0

Naming is based on best blast hit and *P* value based on *n*=3.

**Table 2:** *Litomosoides sigmodontis*-derived miRNAs found in mouse serum.

**Name**	**Mature sequence**	**Number of reads (infected)**
miR-100a	UACCCGUAGCUCCGAAUAUGUGU	479
miR-86	UAAGUGAAUGCUUUGCCACAGUCU	57
Bantam-a	UGAGAUCAUUGUGAAAGCUAUU	45
Bantam-b	UGAGAUCACGUUACAUCCGCCU	45
miR-100b	AACCCGUAGUUUCGAACAUGUGU	40
miR-71	UGAAAGACAUGGGUAGUGAGACG	32
miR-100c	AACCCGUAGAAUUGAAAUCGUGU	22
miR-50-5p	UGAUAUGUCUGAUAUUCUUGGGUU	10
miR-34-5p	UGGCAGUGUGGUUAGCUGGUUGU	8
miR-263/183	AAUGGCACUAGAUGAAUUCACGG	7
Bantam-c	UGAGAUCAUGCCACAUCCGUCU	4
miR-50-3p	CCAGCAUCUCAGACGUAUCGGC	3
miR-153	UUGCAUAGUCACAAAAGUGAUG	3
miR-87-5p	CGCCUGGGACUUCGACUCAACCU	2
miR-2	UAUCACAGCCAGCUUUGAUGU	2
miR-5866	UUACCAUGUUGAUCGAUCUCC	2

miRNA, micro RNA.

miRNAs that map exclusively to the *L. sigmodontis* but not mouse genome, which were identified in the sera of mice infected with *L. sigmodontis* (40 infective larvae were injected subcutaneously and allowed to migrate to the pleural cavity where they developed naturally for 60 days). The lettering of miR-100 and bantam family members is arbitrary.
