# Erratum: Exosomes secreted by nematode parasites transfer small RNAs to mammalian cells and modulate innate immunity

**DOI:** 10.1038/ncomms9772

**Published:** 2015-10-22

**Authors:** Amy H. Buck, Gillian Coakley, Fabio Simbari, Henry J. McSorley, Juan F. Quintana, Thierry Le Bihan, Sujai Kumar, Cei Abreu-Goodger, Marissa Lear, Yvonne Harcus, Alessandro Ceroni, Simon A. Babayan, Mark Blaxter, Alasdair Ivens, Rick M. Maizels

Nature Communications 5: Article number: 548810.1038/ncomms6488 (2014); Published: 11252014; Updated: 10222015.

The affiliation details for Mark Blaxter are incorrect in this Article. The correct affiliation details for this author are given below:

Centre for Immunity, Infection and Evolution, School of Biological Sciences, University of Edinburgh, Edinburgh EH9 3FL, UK.

Institute of Evolutionary Biology, School of Biological Sciences, University of Edinburgh, Edinburgh EH9 3FL, UK.

Edinburgh Genomics, School of Biological Sciences, University of Edinburgh, Edinburgh EH9 3FL, UK.

