# Supplementary material for: Exosomes secreted by nematode parasites transfer small RNAs to mammalian cells and modulate innate immunity
Source: Nat Commun. 2014 Nov 25;5:5488. doi: 10.1038/ncomms6488 (PMC4263141; doi:10.1038/ncomms6488)
Supplement: Supplementary Figures and Tables — Supplementary Figures 1-6 and Supplementary Tables 1-3. [file ncomms6488-s1.pdf]

Supplementary Figure 1

a

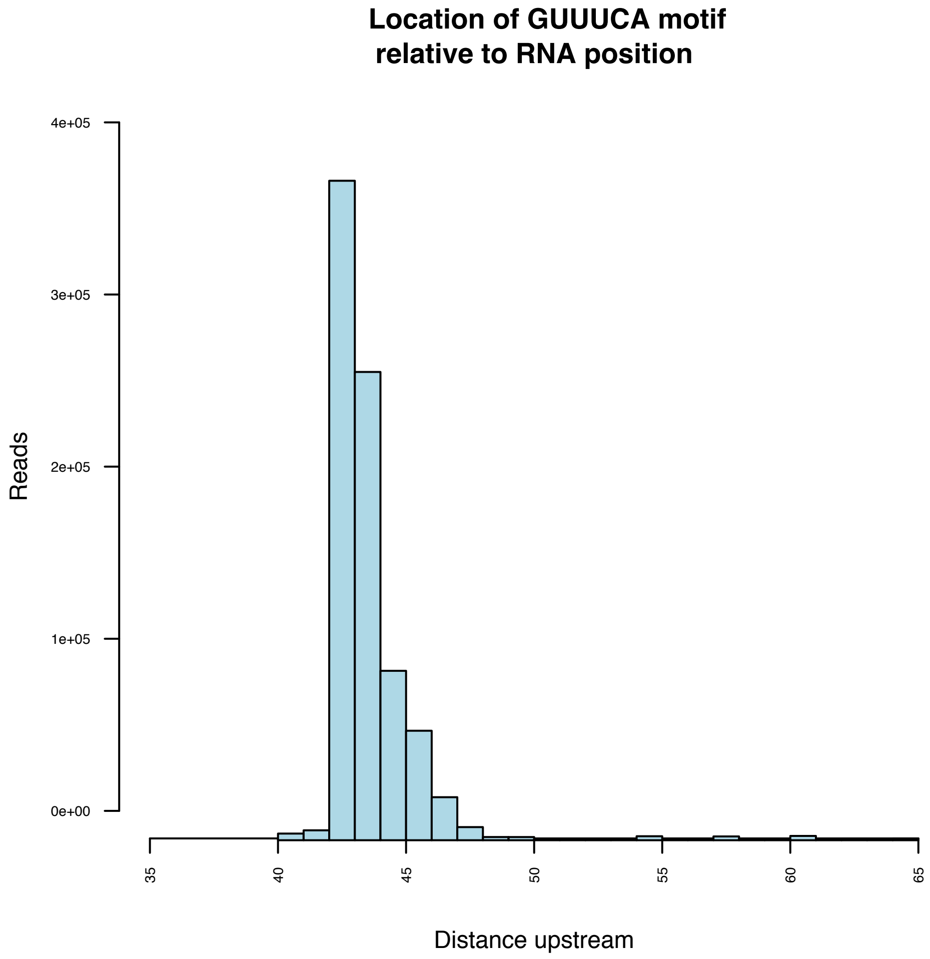

b

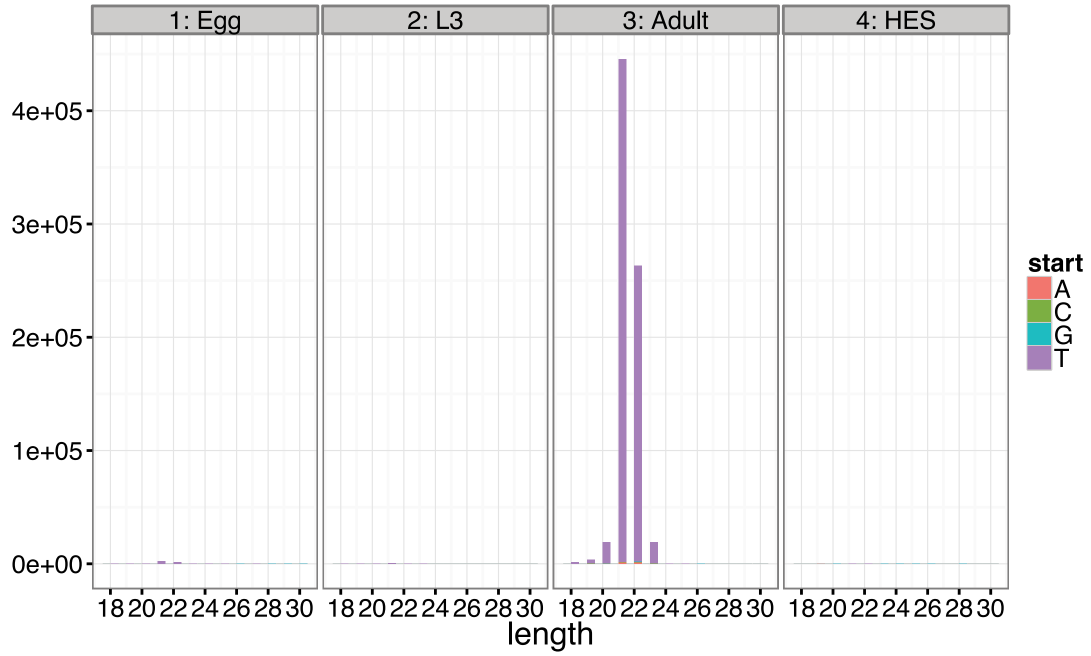

Supplementary Figure 1: piRNAs identified in the adult H.polygyrus library
a, Distribution of “GUUUCA” motif locations upstream of the 5’ RNA alignment site of sequences that were not identified as another class of small RNA (see Methods).
b, Start nucleotide frequency plots for RNA sequences with a "GUUUCA" motif 42-45 nucleotides upstream of the 5' alignment site.

Supplementary Figure 2

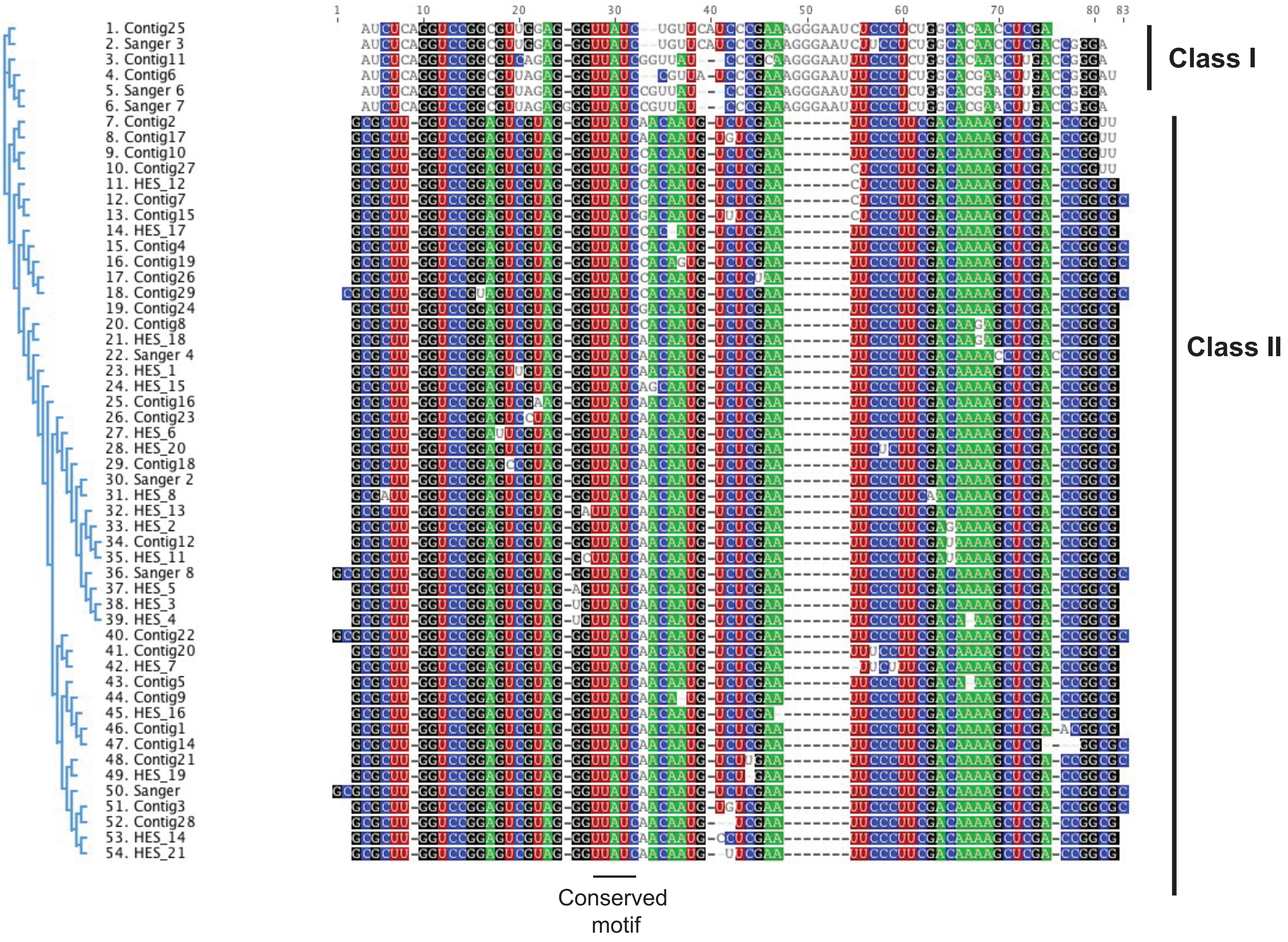

Supplementary Figure 2: Full length nematode Y RNAs identified in the secretion product
Sequence alignment of all Y RNAs identified in the total secretion library based on hairpin structure and “UUAUC motif” in the loop. Given the sequence heterogeneity of the adult worms and incomplete genome, it is not possible to infer whether sequence differences indicate distinct genes or polymorphisms.

Supplementary Figure 3

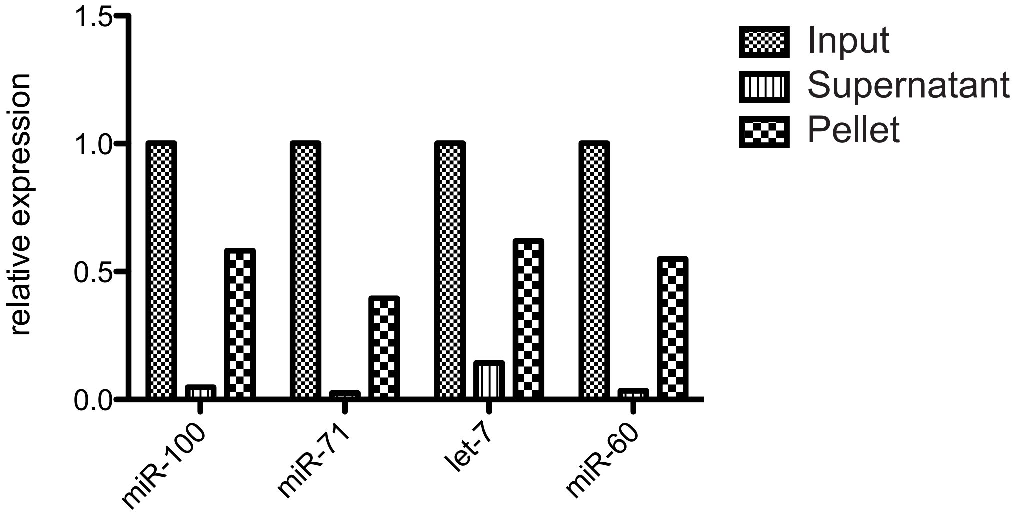

Supplementary Figure 3: Parasite-derived miRNAs from the secretion product pellet upon ultracentrifugation Relative expression is based on qRT-PCR analysis of total RNA extracted from secretion product prior to (“input”) and following ultracentrifugation (“supernatant” and “vesicle fraction”); values were normalized to input secretion products, based on equal volumes of starting material.

Supplementary Figure 4

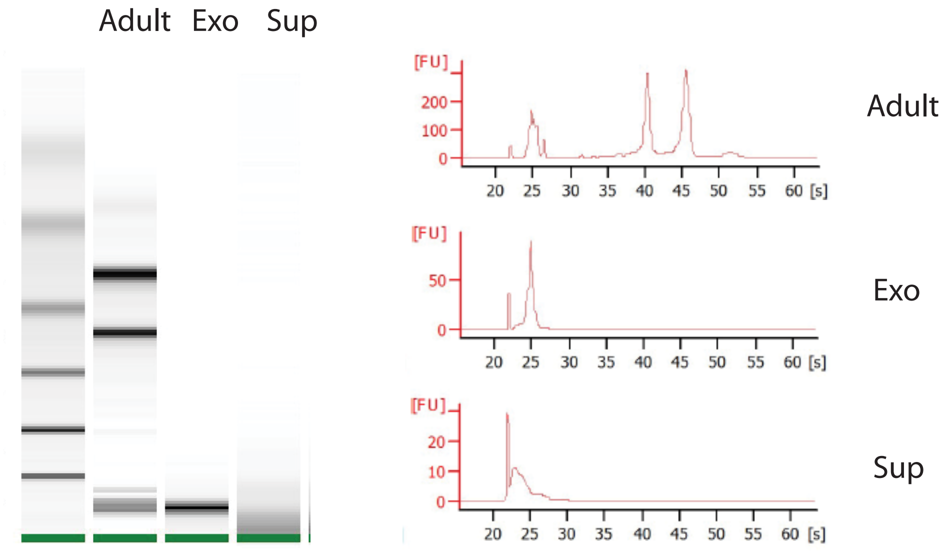

Supplementary Figure 4: Bioanalyzer profile of total RNA extracted from adult *H. polygyrus* compared to vesicle and supernatant fractions of the secretion product.

Supplementary Figure 5

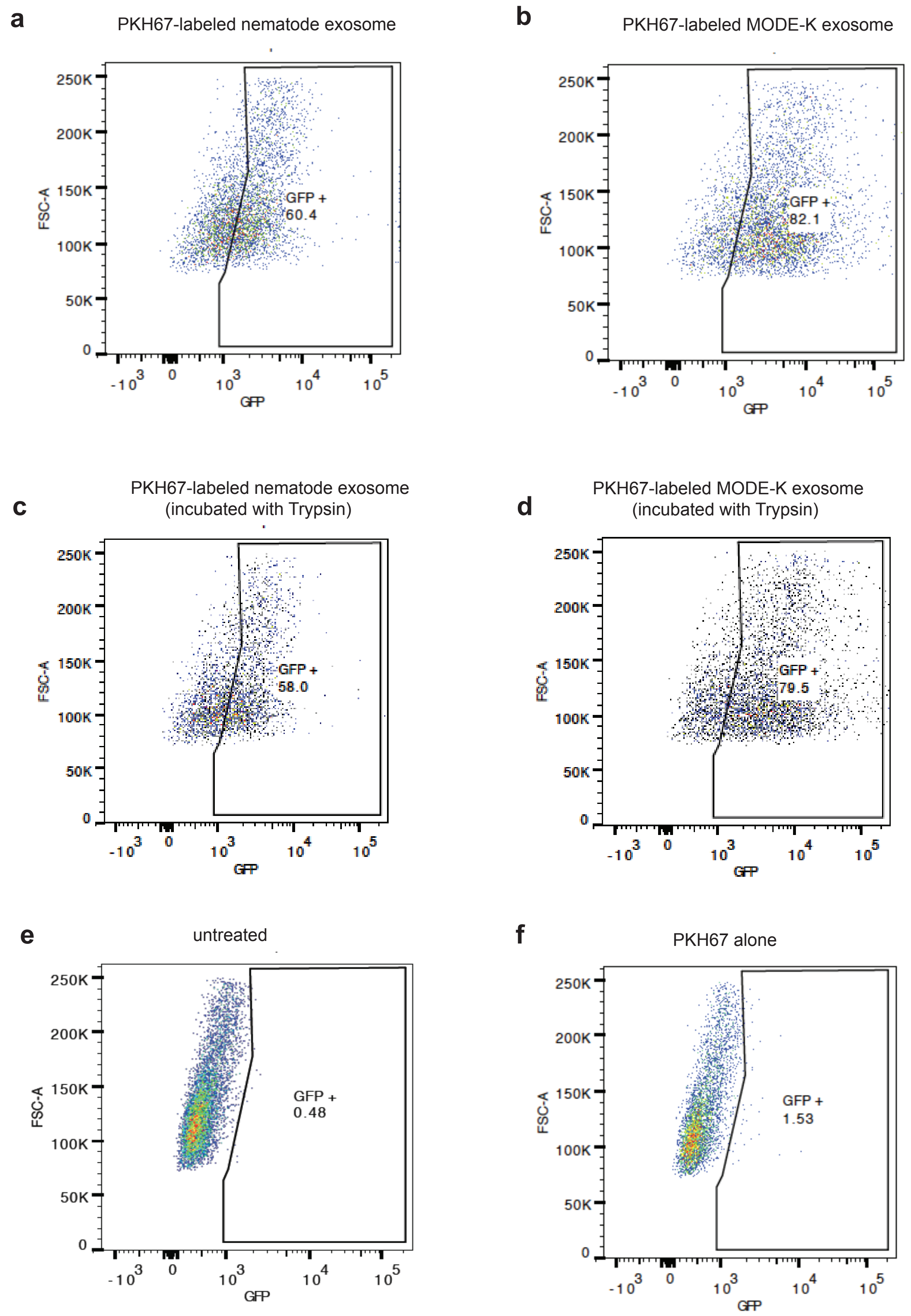

Supplementary Figure 5: Uptake analysis by FACS of H.polygyrus exosomes or MODE-K exosomes into MODE-K cells Cells (1 X 105) were incubated with 5 µg (total protein) nematode (**a**) or MODE-K (**b**) exosomes that were pre-labeled with PKH67 and washed by ultracentrifugation; cells were either directly analyzed by FACs or first pre-incubated with Trypsin for 5 min prior to analysis (**c,d**) to eliminate signal due to non-specific association with the cell surface. The control cells were either untreated (**e**) or incubated with PKH67 prepared the same way in the absence of exosomes (**f**) to control for carry over.

Supplementary Figure 6

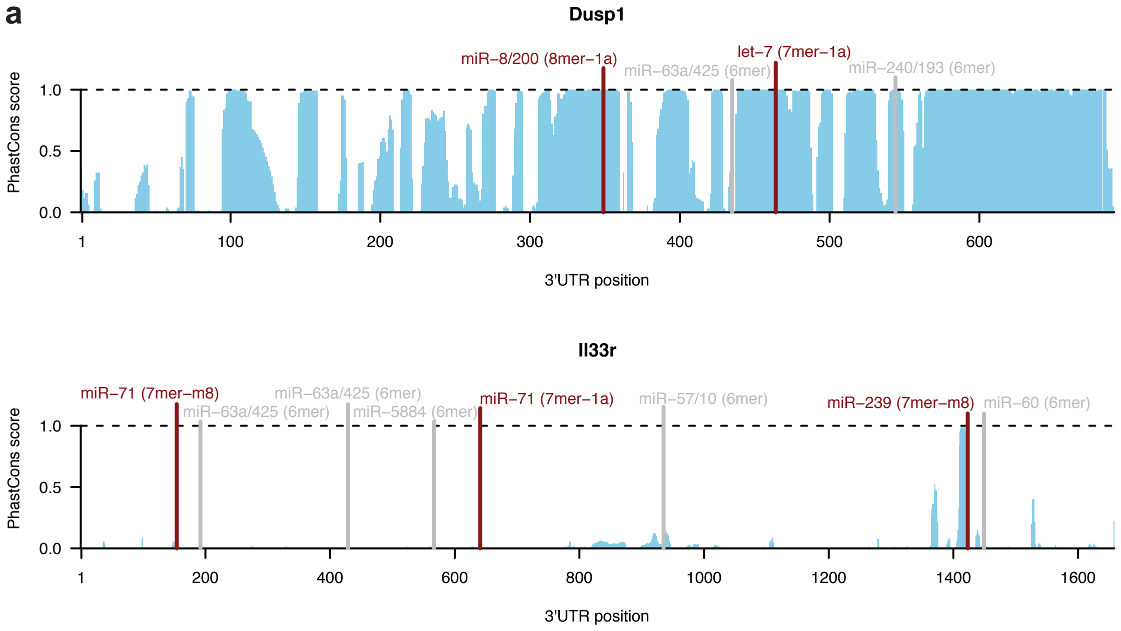

b

miR-200 site

```
5'-UUGACACACCCACCAGUAUUA-3' nt 325 in 3'URT
                ||||||||
3'-GUAGUAAUGGACUGUCAUAAU-5'  hpo-miR-236-3p/miR-200
```

miR-425 site

```
5'-     GCCUUCACAAAUGUCAUU-3' nt 421 in 3'URT
                  ||||||
3'-GGGAAGAGCGUCUUCACAGUAU-5'  hpo-miR-236-3p/miR-200
```

let-7 site 1

```
5'-UCAUAGAAGAACCAAAUACCUCAA-3' nt 444 in 3'UTR
                    |||||||
3'-AGUAUUGUAAAUUUUGAUGGAGU-5'  hpo-let-7
```

Supplementary Figure 6: Conservation and miRNA binding sites of Dusp1 and Il33r 3'UTRs
a, Potential binding sites for *H. polygyrus* microRNAs were identified by searching for seed-matching sites. Genomic coordinates were used to extract PhastCons conservation scores from the 'phastCons60wayPlacental' table of the UCSC Genome Browser. Target types represent perfect seed-matches relative to 5' end of miRNA: 6mer = positions 2-7, 7mer-1a = 2-7 with 'A' across from position 1, 7mer-m8 = 2-8, 8mer-1a = 2-8 with 'A' across from 1. Positions of weak sites (6mers) are indicated in grey, stronger sites (7-8mer) in dark red. **b**, Potential target sites of parasite-derived miRNAs in mouse Dusp1 3'UTR.

Supplementary Table 1: Most abundant nematode miRNAs identified in *H. polygyrus* secretion product libraries

Name	Mature sequence	Illumina V1.5	TrueSeq	
		Total HES RANK	HES vesicles RANK	HES supernatant RANK
miR-100a	AACCCGUAGAUCCGAACUUGUGU	1	8	8
miR-71	UGAAAGACAUGGGUAGUGAGAC	2	15	22
Bantam-a	UGAGAUCAUCACCAUAAGCACA	3	6	12
Bantam-b	UGAGAUCACGCGUAUAUUCGCC	4	3	3
Bantam-c	UGAGAUCAUUGUGAAGGCCACU	5	1	2
miR-100b	UACCCGUAAUGAUAAUGAUCCCGA	6	24	27
miR-100c	UACCCGUAAUGUACAUAGCUUGAG	7	16	20
miR-83/29	UAGCACCAUGUAAAUUCAGCA	8	7	15
miR-60	UAUUAUGCACAUUUUCUGGUUCA	9	28	43
Bantam-d	UGAGAUCACGCCUAUAUUCGCU	10	12	16
Bantam-e	UGAGAUCACGCGUAUAUUCGC	11	14	32
Let-7a	UGAGGUAGUAGGUUGUAUAGUU	12	19	21
miR-63a/425	UAUGACACUUCUGCGAGAAGGG	13	10	18
miR-79/9	AUAAAGCUAGGUUACCAAAGCU	14	42	51
miR-239	UUUGUACUCAGCAAAGAUGCUGC	15	36	46
miR-100d	UACCCGUAUGAGUUUCCGCUGAG	16	35	45
miR-87	UUGAGCAACGAAUAUGUGCGGGA	17	65	69
Let-7b	UGAGGUAGUUUUAAAUGUUAUGA	18	30	33
miR-77	UUCAUCAGGCCUCAGUCAUCCAC	19	26	41
miR-263/183	AAUGGCACUGCAUGAAUUCACGG	20	21	25
**miR-240/miR-193**	**UACUGGCCUCGUAACUUCUAGC**	**24**	**2**	**5**
**miR-57/miR-10**	**UACCCUGUAGUACCGAAUAGUGUCU**	**39**	**4**	**7**
**miR-8/200**	**UAAUACUGUCAGGUAAUGAUGCU**	**33**	**5**	**11**
miR-100g	UACCCGUAGCAUCUGUAUGUCU	47	9	1
miR-239	GACGAAUUCGUCUGGGAUUGGC	26	11	19
miR-63b/miR-425	UAUGACACUGUUGCGAACUGGGAU	27	13	13
lin-4/miR-125	UCCCUGAGACCUCAAUUGCGA	22	17	23
miR-5884	UAGGGUACUGACAUUGAAUGAGUU	44	18	17
miR-44a	UGACUAGAGACACAUUCAGCU	45	20	6
miR-44b	UGACUAGAUCCAUACUCAGCC	50	34	14
miR-44c	UGACUAGACUGUUACUCGGCGU	42	38	10

Relative ranking of miRNAs identified in 2 different library preparations: total HES, prepared by Illumina v1.5 kit (Fig. 1) or the vesicle and non-vesicle fractions (n=3 each), prepared by the TrueSeq kit. miRNAs noted in bold were identified at >10,000 rpm in the Trueseq libraries but not the Illumina v1.5 libraries.

Supplementary Table 2: Percentage of miRNA reads mapping to the listed miRNAs in vesicle libraries from 3 replicates

Name	Rep 1	Rep 2	Rep 3
Bantam-c	45.1%	34.0%	36.4%
Bantam-b	14.4%	11.3%	10.3%
miR-240/miR-193	8.0%	15.9%	20.5%
miR-57/miR-10	5.6%	9.6%	7.8%
miR-8/200	3.7%	3.7%	3.4%
Bantam-a	3.0%	3.5%	3.1%
miR-83/29	1.9%	2.0%	1.6%
miR-100a	2.5%	1.4%	1.6%
miR-100g	1.0%	2.4%	1.7%
miR-63a/425	1.5%	1.8%	1.1%
miR-239	1.5%	1.5%	1.5%
Bantam-d	1.4%	1.6%	1.4%
miR-63b/miR-425	0.7%	1.2%	1.2%
Bantam-e	1.1%	0.6%	0.6%
miR-100c	0.8%	0.5%	0.5%
lin-4/miR-125	0.8%	0.5%	0.5%
miR-71	0.5%	0.7%	0.8%
miR-44a	0.4%	0.7%	0.3%
let-7a	0.5%	0.6%	0.4%
miR-263	0.5%	0.5%	0.4%
miR-5884	0.2%	0.7%	0.8%

The percentages in each library are normalized to the total miRNA reads in that library

Supplementary Table 3: Small RNA classification in serum of mice infected with *L.sigmodontis*

Trimmed reads	23,164,032
Mouse genome match	12,657,071
Unambigious	12,655,769
rRNA	13,563
tRNA	11,342,328
other rfam	11,085
miRNA	1,273,839
L.sigmodontis genome match	2,490
Unambigious	1,188
rRNA	131
tRNA	151
other rfam	18
miRNA	768

Unambiguous reads refer to those which only map to mouse or *L.sigmodontis* genomes (but not both).
